# Quiet Quitting in the Healthcare Workforce: A Systematic Review of Organisational Drivers and Managerial Implications

**DOI:** 10.1155/jonm/7319117

**Published:** 2026-07-22

**Authors:** Elisabetta Cesaro, Chiara Daicampi

**Affiliations:** ^1^ Department of Medicine, University of Padua, Padua, Italy, unipd.it; ^2^ Healthcare Profession Department, Padua University Hospital, Padua, Italy, unipd.it

**Keywords:** burnout, healthcare workers, leadership, occupational stress, organisational support, quiet quitting, work engagement

## Abstract

**Background:**

Quiet quitting refers to employees limiting their efforts to core job responsibilities without engaging in extra‐role activities. Quiet quitting is a relevant social issue, and it represents a deeper pattern of disengagement driven by perceived undervaluation, workload pressures and organisational disconnection. In healthcare, this phenomenon is particularly critical given its potential impact on workforce sustainability, job satisfaction and quality of care.

**Objectives:**

To synthesise evidence on quiet quitting among hospital healthcare workers, with a specific focus on identifying and critically analysing its organisational and occupational drivers, as well as its prevalence and associated sociodemographic factors.

**Methods:**

A systematic search of PubMed, Scopus and EBSCO (CINAHL, PsycINFO, PsycArticles, Psychology and Behavioural) was conducted for quantitative studies published in English between January 2015 and August 2025. Eligible studies assessed quiet quitting in healthcare workers using validated instruments or single‐item indicators. Methodological quality was assessed with the Joanna Briggs Institute checklist. Data were narratively synthesised. The protocol was registered in PROSPERO (CRD420251114378) in August 2025.

**Results:**

Fourteen cross‐sectional studies including 8279 healthcare workers were included. Reported prevalence of quiet quitting ranged from 46.1% to 74.4%. Burnout, job dissatisfaction, work stress, leadership style, organisational support and work environment were consistently associated with higher levels of QQ. Conversely, engaging leadership, supportive environments and higher emotional intelligence were linked to lower quiet quitting. Evidence regarding demographic correlates was heterogeneous; some studies suggested higher prevalence among younger workers, including Generation Z. Most studies were rated as moderate to high quality.

**Conclusions:**

Quiet quitting is common among healthcare workers and appears to be influenced by occupational and organisational factors. Findings should be interpreted with caution due to the cross‐sectional designs, heterogeneous measurement approaches and limited cross‐cultural validation. Future longitudinal and interventional studies should examine whether and how quiet quitting affects workforce sustainability, care quality and patient outcomes across diverse healthcare settings.

**Implications for Nurse Leaders:**

Nurse leaders and managers should address quiet quitting through participatory and empowering leadership, shared governance, professional recognition, organisational support, and fair staffing and workload allocation. Creating psychologically safe and supportive work environments that foster teamwork, well‐being and engagement may strengthen nurses’ sense of belonging, reduce disengagement and support workforce retention and sustainability.

## 1. Introduction

Quiet quitting (QQ) has recently been described as a threshold in employees’ willingness to exert effort during work activity [1]. Employees who engage in QQ behaviours fulfil their formal job responsibilities but are unwilling to assume tasks beyond their defined role [2], consciously avoiding additional duties outside of working hours [3].

In recent years, QQ has emerged as a widely studied phenomenon in the fields of psychology and human resources and has gained popularity through social media and coverage in nonscientific publications and mainstream media. However, Olejniczak‐Szuster [4] suggests that QQ reflects a deeper, value‐driven shift in employee attitudes, rather than a fleeting social media trend; in fact, it represents a silent protest or disengagement from a role or organisation due to feeling undervalued, overworked or disconnected [2].

The phenomenon has been studied in different contexts and among various professions [1, 5–7] given its implications for human resources management and productivity. QQ—limiting one’s efforts to contractual duties—does not necessarily indicate disengagement. Setting boundaries differs from reduced commitment; thus, an employee may quiet quit while remaining engaged or holding a positive or neutral attitude toward their work [1].

Several work‐related factors appear to predict QQ, including occupational stress, burnout, change fatigue [5], lower job embeddedness [8] and perceived leadership style [9]. In contrast, some evidence suggests that QQ is particularly prevalent among Generation Z (individuals born approximately between 1997 and 2012) employees [8, 10], despite their relatively recent entry into the workforce and presumed lower exposure to long‐term stress or burnout.

While numerous studies have examined QQ among workers in the education and hospitality sectors, a comprehensive analysis of QQ among healthcare professionals—summarising its prevalence, characteristics and associated factors—remains lacking. The phenomenon of QQ is important to study in healthcare because it reflects a form of psychological or behavioural disengagement in a sector where professional well‐being, responsiveness and sustained commitment are closely connected to care processes, quality of care and patient safety [11]. While direct evidence linking QQ itself to patient outcomes remains limited, its potential organisational consequences warrant attention in healthcare workforce research. Most notably, the healthcare sector is undergoing substantial changes, with the phenomenon of the ‘Great Resignation’ representing one of the most visible developments [12]. In exploring its underlying causes, healthcare work environments are consistently identified as highly stressful settings [13]. The increasing demand for healthcare services has contributed to longer working hours, elevated stress levels and greater fatigue among healthcare professionals [14]. These pressures are further compounded by adverse working conditions, limited organisational support [15] and, in some cases, authoritarian leadership [16], which collectively exacerbate workforce strain, turnover intentions and physical withdrawal behaviours. In addition, chronic understaffing in healthcare has been shown to negatively affect patient safety [17], whereas adequate staffing, effective teamwork and a balanced workload enable healthcare professionals to better meet patient care needs [18]. Taken together, these global pressures create the organisational conditions in which QQ may emerge as a boundary‐setting or withdrawal response among healthcare workers. In this context, understanding the antecedents of QQ in healthcare may have important implications for workforce sustainability, organisational performance and, potentially, care quality.

The aim of this systematic review is to synthesise the available evidence on the QQ phenomenon among healthcare workers, with a specific focus on identifying and critically analysing its organisational and occupational drivers, as well as its prevalence and associated sociodemographic factors.

## 2. Methods

**FIGURE 1:**
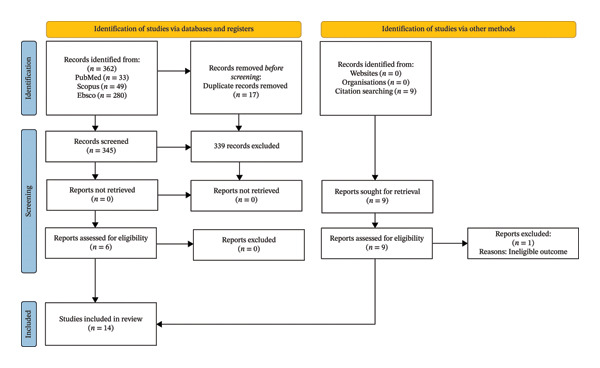
PRISMA 2020 flow diagram for new systematic review which included searches of database, registers and other sources.

The systematic review was guided by the Preferred Reporting Items for Systematic Reviews and Meta‐Analyses (PRISMA) statement [19] provided in Figure 1. A predefined protocol was designed and registered in the international Prospective Register of Systematic Reviews database (PROSPERO) in August 2025 (registration number: CRD420251114378).

### 2.1. Search Strategy

In consultation with two health sciences librarians experienced in systematic review searching, we developed a comprehensive strategy tailored to each database. We searched PubMed, Scopus and the EBSCO platform (CINAHL, APA PsycArticles, Psychology and Behavioural Sciences Collection and APA PsycINFO) for studies published between January 2015 and August 2025. Search terms captured the main concepts of QQ (e.g., ‘quiet quitting’, ‘silent resignation’, ‘workforce disengagement’) and healthcare professionals (e.g., ‘nurses’, ‘healthcare workers’, ‘medical staff’). Boolean operators (‘AND’, ‘OR’) were applied as appropriate. The complete search strings for each database are provided in Supporting Table 1.

The search was carried out in August 2025.

### 2.2. Eligibility Criteria

Eligible studies were primary quantitative investigations focusing on healthcare workers, available in full text, published in English and appearing in a peer‐reviewed indexed journal between January 2015 and August 2025. We excluded qualitative studies, reviews, commentaries, conference abstracts, dissertations and other forms of grey literature. Qualitative research was omitted to ensure comparability of outcomes and enable synthesis of prevalence estimates and statistical associations. The time frame was chosen because QQ is a relatively recent concept, and studies published before 2015 were unlikely to be relevant.

### 2.3. Quality Appraisal

The Joanna Briggs Institute Quality Critical Appraisal Checklist tool for Analytical Cross‐Sectional Studies [20] was used to evaluate the methodological quality and the risk of bias of included studies. The tool consists of eight items with four possible responses: ‘Yes’, ‘No’, ‘Unclear’ or ‘Not applicable’.

Studies were rated as high quality (or low bias) when ≥ 80% of items were satisfied, moderate quality (or moderate bias) when 50%–79% were satisfied, and low quality (or high risk of bias) when < 50% were satisfied. The JBI checklist does not prescribe universal percentage‐based thresholds for overall quality classification; therefore, these cut‐off points were established by the authors for the purposes of this review, in line with commonly used approaches in systematic reviews to guide the interpretation of methodological quality [21]. Two authors independently appraised all studies, and disagreements were resolved by discussion until consensus was reached. The complete evaluation is provided in Supporting Table 2.

### 2.4. Data Extraction and Synthesis

Study selection and data extraction were independently performed by two reviewers using the Rayyan platform. No automation tools were employed at any stage of the process. For each study, reviewers recorded the authors, year, study design, country, setting, population, aim and main findings using a descriptive approach. Any discrepancies or disagreements between reviewers were resolved through discussion and consensus. Findings were synthesised narratively, as provided in Table 1 **.**

**TABLE 1:** Summary of the characteristics of included studies.

Author, year	Study design	Country, setting, population	Aim	Measurement	Main findings	Quality of study
Stankovic, M., et al. 2025	Cross‐sectional	Serbia, Public Health Organizations, n. 647 Healthcare professionals	To investigate how quality of work life dimensions (psychological, physical, cultural, social) influences quiet quitting among healthcare professionals	Quiet Quitting Scale (QQS), Quality of Work Life Questionnaire (covering psychological, physical, social, cultural dimensions).	Poorer quality of work life increased quiet quitting, with gender differences (psychological, physical, and cultural QWL mattered more for women; social well‐being mattered for men).	MODERATE
Galanis, P., et al. 2024	Cross‐sectional	Greece, n.1760 Healthcare workers	To assess quiet quitting levels among healthcare workers and compare nurses with physicians and other HCWs; and to examine how sociodemographic factors, job burnout, and job satisfaction relate to quiet quitting	Quiet Quitting Scale (QQS), Copenhagen Burnout Inventory, Job Satisfaction Survey	Quiet quitting was more prevalent among nurses than among other HCWs, higher burnout and lower job satisfaction were associated with higher quiet quitting. Shift work and employment in the private sector were linked to higher quiet quitting; more clinical experience was associated with lower quiet quitting; higher academic degree related to higher detachment within QQS.	HIGH
Kang, J., et al. 2025	Cross‐sectional	South Korea, General or tertiary hospital, n.323 Nurses	To explore how organisational justice, role ambiguity and job satisfaction mediate the relationship between infection control fatigue and quiet quitting in nurses.	Quiet Quitting Scale (QQS), Infection Control Fatigue Scale, Job Satisfaction Scale, Organisational Justice Scale, Role Ambiguity Scale.	Quiet quitting increased among nurses experiencing infection control fatigue. Job satisfaction and organisational justice mediated this relationship, while role ambiguity did not.	HIGH
Gün, I., et al. 2025	Cross‐sectional	Turkey, n.383 Nurses	To investigate the mediating role of job burnout in the relationship between organisational support and quiet quitting among nurses.	Quiet Quitting Scale (QQS), Perceived Organisational Support Scale, Maslach Burnout Inventory	Organisational support was negatively associated with quiet quitting, while burnout was positively associated. Burnout partially mediated the relationship	MODERATE
Toska, A.; et al. 2025	Cross‐sectional	Greece, General hospital, *n*. 186 Healthcare workers	To explore how workplace conflicts, organisational support and reward systems affect quiet quitting among hospital staff in Greece.	Quiet Quitting Scale (QQS), Questionnaire for Conflicts in Healthcare Organisations, Organisational Support and Reward Fairness items	Quiet quitting was prevalent among hospital staff. Lack of managerial recognition, unfair rewards, and workplace conflicts were key predictors.	HIGH
Ardıç, M., et al. 2025	Cross‐sectional	Turkey, Medical and administrative units, n. 224 Healthcare professionals	To examine the effects of work stress, job satisfaction and work engagement on quiet quitting among healthcare workers	Quiet Quitting Scale (QQS), Job Satisfaction Scale, Work Engagement Scale, Work Stress Scale.	Work stress was positively associated with quiet quitting, while job satisfaction was negatively associated. Work engagement showed only a weak negative association	MODERATE
Galanis, P., et al. 2024	Cross‐sectional	Greece, Healthcare Services, n. 957 Nurses	To investigate the impact of moral resilience on quiet quitting, job burnout, and turnover intention among nurses in the post‐COVID‐19 era.	Quiet Quitting Scale (QQS), revised Rushton Moral Resilience Scale, single‐item burnout measure, turnover intention scale.	Higher moral resilience was associated with lower quiet quitting, job burnout, and turnover intention. Response to moral adversity and moral efficacy were associated with lower scores across quiet quitting dimensions.	MODERATE
Moisoglou, I., et al. 2025	Cross‐sectional	Greece Clinical Environment, n. 404 Nurses	To examine the relationship between engaging leadership, quiet quitting, and work engagement among nurses.	Quiet Quitting Scale (QQS), Engaging Leadership Scale (ELS‐12), Utrecht Work Engagement Scale (UWES‐3)	Engaging leadership was associated with lower levels of quiet quitting.	HIGH
Rinaldi, S., et al., 2025	Cross‐sectional	Italy, Orthopaedic hospital, n. 76 Nurses	To assess the prevalence of quiet quitting among nurses in a Northern Italian hospital and to identify associated factors	Quiet Quitting Scale (QQS)	46.1% of nurses identified as quiet quitters (cut‐off 2.06 on QQS). Higher prevalence among younger nurses and those with fewer years of service	MODERATE
Othman, A. A., et al. 2025	Cross‐sectional	Egypt, University hospital, n. 482 Nurses	To examine the relationships between nurses’ loyalty, intention to leave, quiet quitting, and quiet firing, and to test the mediating role of quiet quitting	Quiet Quitting and Quiet Firing Scale (QQF), Loyalty Scale, Intention to Leave Scale.	Quiet quitting intentions were positively linked to quiet firing and intention to leave and negatively linked to nurses’ loyalty.	MODERATE
Moisoglou, I., et al. 2025	Cross‐sectional	Greece, n. 425 Nurses	To explore how nurses’ work environment influences quiet quitting and work engagement	Quiet Quitting Scale (QQS), Practice Environment Scale of the Nursing Work Index (PES‐NWI, 5 subscales), Utrecht Work Engagement Scale (UWES‐3)	A poorer work environment was associated with higher levels of quiet quitting.	HIGH
Moisoglou, I., et al., 2024	Cross‐sectional	Greece, n. 328 Nurses	To assess the impact of organizational innovation support on quiet quitting, innovative behaviour, and innovation outputs among nurses	Quiet Quitting Scale (QQS), Innovation Support Inventory (ISI), Innovative Behavior Inventory (IBI), Innovation Outputs Scale	Greater innovation support was associated with lower levels of quiet quitting.	HIGH
Galanis, P., et al. 2024	Cross‐sectional	Greece, n. 992 Nurses	To investigate the association between nurses’ emotional intelligence and quiet quitting, turnover intention, and job burnout.	Quiet Quitting Scale (QQS),Trait Emotional Intelligence Questionnaire–Short Form (TEIQue‐SF), Single‐item Burnout Measure, Single‐item Turnover Intention	Emotional intelligence (selfcontrol, emotionality, sociability, well‐being) was negatively associated with quiet quitting.	HIGH
Galanis, P., et al. 2025	Cross‐sectional	Greece, n. 1092 Nurses	To investigate the effect of nurses’ workload on quiet quitting, turnover intention, and job burnout.	Quiet Quitting Scale (QQS), NASA Task Load Index (NASA‐TLX) for workload, Single‐item Burnout Measure, Single‐item Turnover Intention	Higher workload was positively associated with quiet quitting.	HIGH

Reference lists of included articles were also screened to identify additional relevant evidence.

### 2.5. Ethical Considerations

For this systematic review, obtaining an opinion from the Ethics Committee was not required since its nature was considered secondary. The problem formulation process was conducted with meticulous adherence to the principles of clarity, objectivity and precision, aiming to achieve substantial results related to interventions and care within the scope of practice.

## 3. Results

### 3.1. Search Results

The initial database search yielded a total of 362 records: 33 from PubMed, 49 from Scopus and 280 from EBSCO. After removing 17 duplicates, 345 unique records remained and were independently and blindly screened by two reviewers. Following title and abstract screening, 339 records were excluded because they did not meet the predefined eligibility criteria. The most frequent reasons for exclusion at this stage were wrong outcome, wrong study design and wrong population. Six database‐derived reports were therefore assessed for eligibility in full text, and all six met the inclusion criteria. Citation searching of the reference lists of the eligible and full‐text assessed articles identified nine additional records. These nine reports were assessed for eligibility; one was excluded because it did not report an eligible outcome, and eight met the inclusion criteria and were included in the final synthesis. The contribution of citation searching was considered relevant because QQ is an emerging topic in healthcare research, and related studies may be indexed inconsistently or identified through closely connected reference networks. No further eligible records were retrieved from other sources. In total, 14 studies met the inclusion criteria and were included in the final synthesis. Any uncertainties or disagreements regarding study relevance during screening were resolved through discussion and mutual agreement between the reviewers. The study selection process is summarised in Figure 1.

Due to heterogeneity in measurement tools, cut‐offs and reporting formats, quantitative pooling of subgroup effects was not feasible. Instead, subgroup findings are summarised narratively and tabulated for clarity.

### 3.2. Quality Appraisal and Risk of Bias of the Included Studies

Methodological quality of the included cross‐sectional studies ranged from moderate to high. Eight studies [22–29] were rated as high quality (≥ 80% items satisfied) and six [30–35] as moderate quality (50%–79%). None of the studies met the criteria for low quality (< 50%). The main methodological weaknesses related to the limited identification and management of potential confounders, whereas other domains, such as the validity of exposure and outcome measurement and the appropriateness of statistical analyses, were generally well addressed. Overall, the risk of bias was considered acceptable and confidence in the body of evidence moderate to high, although the cross‐sectional design of the included studies reduces the possibility of drawing causal conclusions.

### 3.3. Study Characteristics

Table 1 summarises the key characteristics of the included studies. The included studies have been published between Years 2024 and 2025. All adopted a cross‐sectional design.

### 3.4. Description of the Population

A total of 8279 healthcare workers were analysed across the 14 studies, with sample sizes ranging from 76 to 1760 participants per study. Ten studies included nurses only [22–26, 29–33], whereas four studies involved broader samples of healthcare workers, healthcare professionals or hospital staff [27, 28, 34, 35].

Geographically, eight studies have been carried out in Greece [22–28, 32], one in Italy [30], one in Serbia [35], two in Turkey [33, 34], one in Egypt [31] and one in South Korea [29].

### 3.5. Prevalence of QQ Among Healthcare Workers

Across the included studies, four different tools were applied to assess QQ. Eleven studies used the scale by Galanis et al. [36], and one study [33] applied the QQ scale by Anand et al. [37], while another study [34] applied the QQ scale by Duran et al. [38]; additionally, a further study [31] utilised the QQ and quiet firing scale by Karadas and Çevik [39].

The highest prevalence of QQ was reported in Galanis et al. [26], where 74.4% of the sample were classified as quiet quitters, whereas the lowest was observed [30] with a prevalence of 46.1%.

When considering mean scores obtained with the Galanis Quiet Quitting Scale (threshold for classification as quiet quitter > 2.06), which was the most frequently applied instrument across the included studies, the lowest value was observed in Italy, with a mean score of 2.07 [30], while the highest was reported in Greece, with a mean score of 2.60 [25], as illustrated in Table 2. To provide an overall estimate of QQ levels among nurses, we calculated a pooled weighted mean score across studies using this scale, which yielded a value of 2.51, as a description of the phenomenon. This estimate reflects the contribution of each study according to its sample size and should be interpreted as a descriptive summary of the evidence rather than a formal meta‐analytic effect.

**TABLE 2:** Prevalence of quiet quitting among healthcare workers.

**Authors, year**	**Measurement**		**Mean (SD)**

Gun I., et al. 2025	The quiet quitting scale by Anand		2.62 (0.93)

Ardic M., et al. 2025	Quiet quitting scale by Boz		3.74 (0.84)

Toska A., et al., 2025	The quiet quitting scale	Total score	2.39 (0.588)
		Detachment	2.18 (0.69)
		Lack of initiative	2.40 (0.75)
		Lack of motivation	2.80 (0.98)

Kang J., et al., 2025	The quiet quitting scale	Total score	2.58 (0.51)

Galanis P., et al. 2024	The quiet quitting scale	Total score	2.43 (0.67)
		Detachment	2.20 (0.74)
		Lack of initiative	2.40 (0.86)
		Lack of motivation	2.91 (0.94)
		% of quiet quitters	71.9%

Rinaldi S., et al. 2025	The quiet quitting scale	Total score	2.07 (0.67)
		Detachment	1.93 (0.71)
		Lack of initiative	2.03 (0.86)
		Lack of motivation	2.40 (1.14)
		% of quiet quitters	46.1%

Moisoglou I., et al. 2024	The quiet quitting scale	Lack of motivation	2.90 (1.03)
		Detachment	2.00 (0.73)
		Lack of initiative	2.39 (0.85)

Moisoglou I., et al. 2025	The quiet quitting scale	Detachment	2.14 (0.78)
		Lack of initiative	2.38 (0.92)
		Lack of motivation	2.94 (1.00)
		% of quiet quitters	66.6%

Moisoglou I, et al. 2025	The quiet quitting scale	Total score	2.40 (0.73)
		% of quiet quitters	66.1%

Galanis P., et al. 2024	The quiet quitting scale	Total score	2.55 (0.74)
		Detachment	2.35 (0.82)
		Lack of initiative	2.52 (0.92)
		Lack of motivation	3.02 (0.94)
		% of quiet quitters	74.4%

Galanis P., et al. 2025	The quiet quitting scale	Total score	2.60 (0.7)

Othman, A. A, et al. 2025	The quiet quitting and quiet firing scale	Quiet quitting Intentions	3.047 (0.991)
		Quiet quitting and quiet firing scale	3.037 (0.957)

Galanis P., et al., 2024	The quiet quitting scale	Total score for nurses	2.36 (0.66)
		Detachment for nurses	2.07 (0.73)
		Lack of initiative for nurses	2.35 (0.88)
		Lack of motivation for nurses	2.96 (0.98)
		% of quiet quitters	67.4%

### 3.6. QQ and Demographic Correlations

As illustrated in Table 3, the relationship between QQ and sociodemographic variables was heterogeneous across studies [23, 27–30, 34]. Age was generally negatively associated with QQ, suggesting that younger healthcare workers were more likely to disengage. This pattern was reported in Italian and Greek studies and was also observed in the Korean study, where age was negatively correlated with QQ [23, 29, 30].

**TABLE 3:** Quiet quitting and demographic variables.

Authors, year	Demographic variable		Quiet quitters	Non‐quiet quitters	Pearson correlation coefficient
Rinaldi S., et al., 2025	Age	Mean (SD)	35.4 (7.4)	39.9 (9.1)	−0.34
	Years of work experience mean		10.9 (7.5)	14.7 (9.7)	−0.32

Moisoglou I., et al., 2025		Adjusted Coefficient Beta (95%CI)			*p* value
	Males’ vs females			0.240 (0.040; 0.440)	0.019
	Age			−0.009 (−0.018; −0.0003)	0.042
	Understaffed ward			0.240 (0.045; 0.435)	0.016
	Shift work			0.047 (−0.106; 0.200)	0.547
	Work experience			0.001 (−0.005; 0.007)	0.754

Ardic M., et al., 2025	**Gender**: Female vs male	Quiet quitting Mean (SD)		3.66 (0.76) vs 4.04 (0.93)	0.001
	**Marital status**: Married vs single			3.95 (0.93) vs 3.59 (0.93)	0.002
	**Working unit**: Medical vs administrative			3.91 (0.83) vs 3.58 (0.82)	0.005
	**Years of experience**: 0–5 vs 6–11 vs ≥ 12			3.63 (0.92) vs 3.78 (0.92) vs 4.00 (0.62)	0.018
	**Educational status**: High school vs bachelor vs postgraduate			3.70 (0.96) vs 3.78 (0.78) vs 3.99 (0.75).	0.209

Kang J., et al., 2025	**Gender**: Female vs male	Quiet quitting mean (SD)		2.70 (0.57) vs 2.56 (0.50)	0.13
	**Educational level:** Associate degree vs bachelor’s degree vs graduate school or above			2.63 (0.53) vs 2.63 (0.51) vs (2.42 (0.49)	< 0.01
	**Hospital type:** Tertiary hospital vs general hospital			2.52 (0.47) vs 2.65 (0.56)	< 0.01
	**Position:** General nurse vs charge nurse vs advanced practice nurse.			2.64 (0.52) vs 2.51 (0.49) vs 2.29 (0.40)	< 0.01

Galanis P., et al., 2024	**Professional profile:** Nurses vs physician vs other healthcare workers	Quiet quitting mean (SD)		2.36 (0.66) vs 2.06 (0.65) vs 2.05 (0.58)	< 0.001

Work experience showed a partly similar but not fully consistent pattern. Fewer years of experience were associated with higher QQ in the Italian study, and negative associations with clinical or total work experience were also reported in the Greek and Korean studies [27, 29, 30]. In contrast, Ardıç and Erişen reported higher QQ scores among healthcare workers with more years of experience, while Moisoglou et al. did not find a significant adjusted association between work experience and QQ [23, 34]. Gender‐related findings were inconsistent. Moisoglou et al. and Ardıç and Erişen reported higher QQ among males, whereas Kang et al. reported higher mean QQ scores among females, although this difference was not statistically significant [23, 29, 34]. Marital status appeared relevant in one study, with married workers reporting higher QQ scores than single workers [34].

Educational level showed heterogeneous associations across studies. Galanis et al. reported higher detachment scores among healthcare workers with an MSc/PhD diploma, whereas Kang et al. reported lower mean QQ scores among nurses with graduate‐level education, and Ardıç and Erişen did not find a significant difference according to educational status [27, 29, 34].

Finally, occupational characteristics were found to play a role. Nurses were more likely to report higher levels of QQ compared with physicians and other healthcare workers [27]. Similarly, employees working in medical rather than administrative units [34], in general rather than tertiary hospitals [29], on rotating shifts [27] or in understaffed wards [23] were all more likely to exhibit QQ behaviours.

### 3.7. QQ and Other Work‐Related Variables

Several work‐related variables were associated with QQ, assessed either through validated multi‐item scales or single‐item indicators, as provided in Table 4.

**TABLE 4:** Quiet quitting and work‐related variables.

Authors, year	Outcome	Measurement	Correlation		Adjusted coefficient beta (95% CI)	*p* value
Moisoglou I., et al. 2024	To examine the impact of innovation support on quiet quitting	The quiet quitting scale and innovation support inventory (ISI)	Detachment	Managerial support	−0.173 (−0.281; −0.065)	0.002
				Organizational support	0.012 (−0.132; 0.189)	0.563
				Cultural support	−0.036 (−0.159; 0.087)	0.565
			Lack of initiative	Managerial support	−0.314 (−0.435; −0.194)	< 0.001
				Organizational support	−0.038 (−0.167; 0.090)	0.560
				Cultural support	−0.037 (−0.175; 0.100)	0.592
			Lack of motivation	Managerial support	−0.331 (−0.469; −0.192)	< 0.001
				Organizational support	−0.187 (−0.334; −0.039)	0.014
				Cultural support	−0.288 (−0.446; −0.130)	< 0.001

Moisoglou I., et al. 2025	To examine the effect of nurses ‘work environments on quiet quitting and work engagement	The quiet quitting scale and Practice Environment Scale – 5 (PES‐5)	Nurse participation in hospital affairs		−0.116 (−0.218; −0.014)	0.026
			Nurse manager ability, leadership and support		−0.177 (−0.259; −0.095)	< 0.001
			Collegial nurse–physician relationship		−0.134 (−0.232; −0.037)	0.007
			Staffing and resource adequacy		0.042 (−0.060; 0.144)	0.415
			Nursing foundations for quality of care		−0.133 (−0.229; −0.038)	0.006

Galanis P., et al., 2024	Investigate the impact of moral resilience on quiet quitting, job burnout, and turnover intention in nurses.	The Quiet Quitting Scale, revised Rushton Moral Resilience Scale, single‐item burnout measure, turnover intention scale.	Detachment	Response to moral adversity	−0.11 (−0.18; −0.03)	0.005
				Personal integrity	−0.10 (−0.20; −0.002)	0.046
				Relational integrity	−0.15 (−0.24; −0.06)	0.0002
				Moral efficacy	−0.22 (−0.34; −0.11)	< 0.001
			Lack of initiative	Response to moral adversity	−0.15 (−0.23; −0.06)	0.001
				Personal integrity	−0.04 (−0.15; 0.07)	0.44
				Relational integrity	−0.18 (−0.28; −0.08)	0.001
				Moral efficacy	−0.50 (−0.63; −0.37)	< 0.001
			Lack of motivation	Response to moral adversity	−0.31 (−0.41; −0.22)	< 0.001
				Personal integrity	−0.06 (−0.19; 0.07)	0.35
				Relational integrity	−0.01 (−0.13; 0.11)	0.89
				Moral efficacy	−0.32 (−0.47; −0.17)	< 0.001

Moisoglou I., et al. 2025	To examine the impact of engaging leadership on quiet quitting	The quiet quitting scale and Engaging Leadership Scale −12	Detachment	Strengthening	0.043 (−0.105; 0.192)	0.566
				Connecting	−0.215 (−0.365; −0.065)	0.005
				Empowering	0.095 (−0.038; 0.228)	0.160
				Inspiring	−0.146 (−0.289; −0.004)	0.043
			Lack of initiative	Strengthening	−0.060 (−0.223; 0.104)	0.473
				Connecting	−0.199 (−0.364; −0.034)	0.018
				Empowering	−0.002 (−0.148; 0.144)	0.978
				Inspiring	−0.106 (−0.263; 0.050)	0.483
			Lack of motivation	Strengthening	−0.078 (−0.267; 0.111)	0.418
				Connecting	−0.042 (−0.233; 0.149)	0.665
				Empowering	−0.019 (−0.189; 0.150)	0.825
				Inspiring	−0.233 (−0.415; −0.052)	0.012

Galanis P., et al. 2024	To examine the relationship between emotional intelligence and quiet quitting	The quiet quitting scale and the Trait Emotional Intelligence Questionnaire‐Short Form	Detachment	Well‐being	−0.05 (−0.11; 0.01)	0.116
				Self‐control	−0.16 (−0.23; −0.09)	< 0.001
				Emotionality	−0.18 (−0.25; −0.10)	< 0.001
				Sociability	−0.02 (−0.08; 0.04)	0.512
			Lack of initiative	Well‐being	−0.07 (−0.14; −0.004)	0.038
				Self‐control	−0.07 (−0.14; −0.001)	0.055
				Emotionality	−0.18 (−0.26; −0.10)	< 0.001
				Sociability	−0.21 (−0.27; −0.15)	< 0.001
			Lack of motivation	Well‐being	−0.19 (−0.26; −0.120)	< 0.001
				Self‐control	−0.09 (−0.17; −0.02)	0.017
				Emotionality	−0.11 (−0.19; −0.03)	0.007
				Sociability	−0.10 (−0.17; −0.04)	0.002

Stankovic M., et al., 2025	To investigate the impact of quality of work life on the indication of quiet quitting.	The quiet quitting scale and the Quality of Work Life Questionnaire		Psychological quality of work life and Quiet quitting	−0.307 (−0.396; −0.212)	< 0.001
				Physical quality of work life and Quiet quitting	−0.136 (−0.230; −0.037)	0.006
				Social quality of work life and Quiet quitting	−0.100 (−0.206; 0.002)	ns
				Cultural quality of work life and Quiet quitting	−0.247 (−0.330; −0.162)	< 0.001

Galanis P., et al., 2025	To evaluate the effect of workload on quiet quitting	The quiet quitting scale and the NASA task load index	Workload		0.009 (0.006; 0.012)	< 0.001

Galanis P., et al., 2024	To assess quiet quitting levels among healthcare workers and compare nurses with physicians and other HCWs.	The quiet quitting scale, the Job Satisfaction Survey, the Copenhagen Burnout Inventory		Shift work and quiet quitting	0.108 (0.047; 0.170)	0.001
				Job in the public sector and quiet quitting and quiet quitting	−0.049 (−0.120; 0.023)	0.180
				Understaffed workplace and quiet quitting	−0.029 (−0.107; 0.049)	0.462
				Years of clinical experience and quiet quitting	−0.009 (−0.012; −0.006)	< 0.001
				Work‐related burnout score and quiet quitting	0.010 (0.008; 0.011)	< 0.001
				Satisfaction score and quiet quitting.	−0.004 (−0.005; −0.003)	< 0.001

				**Variable correlated**	**R(Pearson)**	**p** **value**

Othman A. A, et al., 2025	To examine the relationship between nurses’ loyalty, intention to leave, quiet quitting, and quiet firing	Quiet quitting intentions	Quiet quitting and quiet firing scale	Perceived quiet firing	0.460	< 0.001
				Intention to leave	0.464	< 0.001
				Loyalty	−0.300	< 0.001
		Quiet quitting and quiet firing total score	Quiet quitting and quiet firing scale	Intention to leave	−0.450	< 0.001
				Loyalty	−0.198	< 0.001
		Perceived quiet firing	Quiet quitting and quiet firing scale	Intention to leave	0.450	< 0.001
				Loyalty	−0.289	< 0.001

Gun I., et al., 2025	To explore the mediating role of job burnout in the relationship between organisational support with quiet quitting	The quiet quitting scale by Anand, the Organisational support Scale and the Job Burnout Scale		Organizational support and quiet quitting	−0.519	< 0.001
				Job burnout and quiet quitting	0.628	< 0.001

Ardic M., et al., 2025	To examine the effect of work stress, job satisfaction and work engagement on quiet quitting	Quiet quitting scale by Boz, Work stress scale, Job satisfaction scale, UWES‐3	The Work Stress scale	Work stress and quiet quitting	0.543	< 0.001
			The job satisfaction scale	Job satisfaction and quiet quitting	−0.562	< 0.001
			The Work engagement scale	Work Engagement and quiet quitting	−0.302	< 0.001

Toska A., et al., 2025	To Investigate the correlation between workplace conflicts and Quiet Quitting	The quiet quitting scale and Questionnaire for Conflicts in Healthcare Organizations		Messages clearly understood/shared expectations and quiet quitting	−0.232	< 0.001

Burnout and job satisfaction were among the most frequently assessed parameters in the studies included. Burnout, evaluated using the Job Burnout Scale and the Copenhagen Burnout Inventory, showed a strong positive association with QQ [27, 33].

In contrast, job satisfaction—assessed with the Job Satisfaction Scale, the Job Satisfaction Survey and the Satisfaction Scale for Clinical Nurses—was generally negatively related to QQ, indicating a protective effect [27, 34]. However, one study reported a positive correlation [29], possibly due to reverse scoring of the instrument.

Organisational and psychosocial factors also emerged as significant correlates. Perceived organisational support was consistently reported as protective [33], while organisational justice similarly reduced the likelihood of disengagement [29]. Conversely, workload was identified as a risk factor, contributing to higher levels of QQ [25].

At the individual level, protective associations were observed with quality of work life [35], loyalty [31], emotional intelligence—particularly emotionality, sociability and self‐control [26]—and moral efficacy as a part of moral resilience [32].

Leadership factors were also strongly linked with QQ. Perception of management awareness was found to be protective [28]. Moreover, engaging leadership behaviours, such as connecting and inspiring, were negatively associated with QQ [22], while managerial support and leadership ability also emerged as protective dimensions [23, 24].

Finally, several negative organisational stressors were identified as risk factors. These included work stress [34], perceived quiet firing [31], infection control‐associated fatigue and role ambiguity [29], which were positively associated with QQ, although role ambiguity did not mediate the relationship between infection control‐associated fatigue and QQ.

## 4. Discussion

This review represents one of the first syntheses examining the association between work‐related and personal characteristics and QQ among healthcare workers. Fourteen quantitative studies were included. Although the term QQ is relatively recent, the phenomenon reflects a longstanding behavioural response: When exposed to persistent stressors, individuals may disengage or reduce effort [40].

Within healthcare, QQ may represent a form of resistance to unfavourable organisational conditions rather than a mere lack of motivation, and it can serve as a way for healthcare workers to express opposition to workplace adversities [41].

The findings suggest that QQ is associated with a wide range of sociodemographic, occupational and psychosocial factors.

Results regarding demographic correlates were inconsistent. Several studies suggested higher QQ among younger or less experienced healthcare workers, although not all findings were consistent across contexts [23, 27, 29, 30, 34]. Gender‐ and educational‐level differences were also heterogeneous and should be interpreted cautiously [23, 27, 29, 34]. Such discrepancies may reflect both methodological differences, including the measurement of QQ, and contextual influences, such as cultural norms, professional roles and healthcare system structures.

Similar variability emerged with respect to job satisfaction: While most studies indicated a protective effect [27, 34], one study reported the opposite pattern [29], possibly due to reverse scoring or contextual factors. Supporting this interpretation, higher education was shown to provide more job resources (e.g., income, autonomy and task variety) but also greater demands (e.g., long working hours, workload and intensity), which may offset potential gains and result in lower overall job satisfaction [42].

Role strain and conflict, which have been linked to QQ [29], are important contributors to nurse burnout [43]. Burnout, in turn, has been associated with a poorer safety climate and culture, lower safety grades and higher rates of nosocomial infections, patient falls, medication errors, adverse events and missed or unfinished care [44]. In addition, negative emotional responses among healthcare staff may impair work performance, reduce care quality, increase defensive practices and negatively affect patient safety, including greater use of physical restraint [45]. However, these negative outcomes have not yet been examined in relation to QQ. Although direct empirical evidence linking QQ to patient outcomes is currently lacking, behaviours typically associated with QQ—such as reduced discretionary effort, lower proactive engagement and limited involvement in extra‐role activities [3]—may have indirect implications for care quality, patient safety and continuity of care. These assumptions should be interpreted with caution and warrant further investigation in future studies.

Moreover, evidence indicates that both men and women are equally susceptible to burnout [46], which has emerged as a significant risk factor in our studies [27, 33]. This is particularly evident among younger Generation Z workers [47] who tend to value work–life balance and moderate work intensity [48]. Arvaniti [41] further observed that new staff initially displayed enthusiasm but gradually disengaged after being exposed to colleagues with low motivation, underlining the importance of workplace culture. Organisational factors were also consistently associated with QQ. Empowering leadership and a supportive organisational culture were found to increase job satisfaction, while work overload reduced it [49]. In line with this, studies included in the review confirmed the influence of these dimensions on QQ [22, 29, 34]. Evidence from school settings suggests that democratic leadership, as opposed to autocratic styles, may play a protective role [9]. Taken together, these findings highlight the multifactorial nature of QQ and underscore the importance for healthcare organisations to foster supportive and engaging environments that promote both professional and personal well‐being [50].

### 4.1. Implications for Nursing Management

From a managerial perspective, fostering participatory and empowering leadership, strengthening recognition mechanisms and ensuring a fair distribution of workload may help mitigate QQ behaviours and support workforce sustainability. As QQ has been described as a coping response to adverse working conditions, psychological strain and unmet expectations, manifested through subtle forms of behavioural withdrawal while employees remain physically present at work [51], interventions targeting the work environment appear particularly relevant.

Promoting nurses’ involvement in decision‐making through shared governance may enhance reciprocal relationships, collective ownership and organisational embeddedness, thereby strengthening engagement and reducing the risk of disengagement behaviours [52]. Likewise, the development of supportive work environments that prioritise psychological safety, teamwork and professional development may contribute to greater employee engagement and well‐being.

Evidence suggests that nurse managers’ empowering behaviours are positively associated with healthcare workers’ work engagement [53]. Therefore, adopting empowering leadership practices may represent an effective strategy for enhancing engagement and improving organisational outcomes [54]. In addition, healthcare organisations should implement comprehensive support systems that address occupational challenges, promote well‐being and stress management, and reinforce employees’ sense of belonging and commitment [55].

Team collaboration and professional recognition have also been shown to buffer the negative effects of work overload [56]. Consequently, when immediate reductions in workload are not feasible, nurse managers should prioritise strengthening teamwork and acknowledging healthcare workers’ contributions and professional value to sustain engagement and reduce the likelihood of QQ behaviours. Furthermore, thoughtfully designed and sustainable healthcare facilities may contribute to staff well‐being, recruitment, retention and performance [57]. Although the association between QQ and turnover intention appears to decrease over time [58], preventing disengagement remains essential for maintaining a motivated and sustainable nursing workforce.

### 4.2. Limitations

This review has several limitations. All included studies adopted a cross‐sectional design, preventing causal inference. Consistent weaknesses emerged in the identification and management of potential confounders, as highlighted in the risk of bias assessment, which should be taken into account when interpreting the findings. Different instruments were used to assess QQ, complicating direct comparisons across studies. Moreover, QQ and related work variables were not consistently analysed in relation to demographic characteristics, and results were not stratified by professional groups (e.g., physicians, nurses, administrative staff), limiting the exploration of role‐specific differences. Finally, most studies were conducted in Europe and Asia, reducing the generalisability of findings to other regions. The exclusion of qualitative research may have limited the understanding of the contextual and motivational dimensions underlying QQ.

Taken together, these methodological weaknesses should be considered when interpreting the findings and highlight the importance of conducting more rigorous study designs in the future.

## 5. Conclusion

In conclusion, the phenomenon of QQ among healthcare workers has been explored through various approaches, yet evidence remains limited to a few settings. Burnout shows a strong association with the phenomenon, while organisational factors such as leadership and work environment represent key areas where interventions can mitigate disengagement. Generation Z workers, with many potential years ahead in the workforce, appear particularly susceptible, underscoring the need for healthcare policies that prioritise work–life balance and supportive organisational cultures.

Future studies should adopt longitudinal or interventional designs to clarify the causal mechanism underlying QQ and to test strategies aimed at reducing disengagement.

Future research should also examine whether and how QQ may affect patient safety, quality of care and staff retention.

Finally, studies differentiating between healthcare professions are warranted to identify role‐specific vulnerabilities and inform targeted interventions.

## Author Contributions

Elisabetta Cesaro and Chiara Daicampi made substantial contributions to the conception and design of the review, search strategy development, study selection, data extraction, methodological quality appraisal, analysis and interpretation of the findings, and drafting and critical revision of the manuscript.

## Funding

This research did not receive any grant from funding agencies in the public, commercial or not‐for‐profit sectors. Open access publishing facilitated by Universita degli Studi di Padova, as part of the Wiley ‐ CRUI‐CARE agreement.

## Disclosure

All authors meet the criteria for authorship, and all authors approved the final version of the manuscript and agree to be accountable for all aspects of the work.

## Ethics Statement

This systematic review did not require ethical approval, as it was based exclusively on previously published studies and did not involve human participants, biological samples, personal data collection or primary data.

## Conflicts of Interest

The authors declare no conflicts of interest.

## Supporting Information

Additional supporting information can be found online in the Supporting Information section.

## Supporting information


**Supporting Information 1** Supporting Table 1. Search strategies.

**Supporting Information 2** Supporting Table 2. Methodological quality of the cross‐sectional studies.

## Data Availability

No new primary data were generated for this systematic review. The data supporting the findings of this study are derived from previously published studies cited in the reference list and are summarised in the main tables and supporting information. Additional details on the data extraction and quality appraisal process are available from the corresponding author upon reasonable request.
