# Supplementary material for: Quiet Quitting in the Healthcare Workforce: A Systematic Review of Organisational Drivers and Managerial Implications
Source: J Nurs Manag. 2026 Jul 22;2026:7319117. doi: 10.1155/jonm/7319117 (PMC13392511; doi:10.1155/jonm/7319117)
Supplement: Supplementary file 1 — Supporting Information 1 Supporting Table 1. Search strategies. [file JONM-2026-7319117-s001.docx]

| **Database** | **Search rules** |
| --- | --- |
| **MEDLINE** | ("quiet quitting" OR "silent resignation" OR "resenteeism" OR "naked quitting" OR "loud quitting" OR "workforce disengagement" OR "personnel retention" OR "Voluntary resignation” OR “Voluntary termination” OR "Voluntary quiet”) AND ("Health Personnel"[Mesh] OR "Personnel Health" OR "Healthcare Workers" OR "Healthcare Worker" OR "Health Care Providers" OR "Health Care Provider" OR "Provider Health Care" OR "Healthcare Providers" OR "Healthcare Provider" OR "Provider Healthcare" OR "Health Care Professionals" OR "Health Care Professional" OR "Professional Health Care" OR "hospital staff" OR "medical professionals" OR "healthcare workers" OR "hospital staff"OR "medical professionals" OR "healthcare professionals") |
| **SCOPUS** | ("quiet quitting" OR "silent resignation" OR "resenteeism" OR "naked quitting" OR "loud quitting" OR "workforce disengagement" OR "personnel retention" OR "Voluntary resignation" OR "Voluntary termination" OR "Voluntary quiet") AND ("Health Personnel" [Mesh] OR "Personnel Health" OR "Healthcare Workers" OR "Healthcare Worker" OR "Health Care Providers" OR "Health Care Provider" OR "Provider Health Care" OR "Healthcare Providers" OR "Healthcare Provider" OR "Provider Healthcare" OR "Health Care Professionals" OR "Health Care Professional" OR "Professional Health Care" OR "hospital staff" OR "medical professionals" OR "healthcare workers" OR "hospital staff"OR "medical professionals" OR "healthcare professionals") |
| **EBSCO** | ("quiet quitting" OR "silent resignation" OR presenteeism OR "workforce disengagement" OR "personnel retention" OR "Voluntary resignation" OR "Voluntary termination" OR "Voluntary quiet") AND ("Medical personnel" OR "healthcare workers" OR "hospital staff" OR "medical professionals" OR "healthcare professionals") |

**Supplemental Table 1**. Search strategies
