# Supplementary material for: Quiet Quitting in the Healthcare Workforce: A Systematic Review of Organisational Drivers and Managerial Implications
Source: J Nurs Manag. 2026 Jul 22;2026:7319117. doi: 10.1155/jonm/7319117 (PMC13392511; doi:10.1155/jonm/7319117)
Supplement: Supplementary file 2 — Supporting Information 2 Supporting Table 2. Methodological quality of the cross‐sectional studies. [file JONM-2026-7319117-s002.docx]

| **Criterion** | **Stankovic & Slavkovic, 2025** | **Gun et al., 2025** | **Rinaldi S., Pomarolli E. 2025** | **Othman A.A. et al.**  **2025** | **Moisoglou I. et al., 2024** | **Moisoglou I. et al., 2025** | **Moisoglou I. et al., 2025** | **Galanis p. et al., 2024** | **Galanis P. et al., 2025** | **Ardıç & Erişen, 2025** | **Toska, Dimitriadou et al., 2025** | **Galanis, Moisoglou, Katsiroumpa et al., 2024** | **Galanis et al., 2024.** | **Kang, J., Jeong, W. & Kim, S., 2025** |
| --- | --- | --- | --- | --- | --- | --- | --- | --- | --- | --- | --- | --- | --- | --- |
| **1.Were the criteria for inclusion in the sample clearly defined?** | **+** | **-** | **+** | **+** | **+** | **+** | **+** | **+** | **+** | **+** | **+** | **-** | **+** | **+** |
| **2. Were the study subjects and the setting described in detail?** | **-** | **-** | **+** | **+** | **+** | **+** | **+** | **+** | **+** | **-** | **+** | **-** | **-** | **+** |
| **3.Was the exposure measured in a valid and reliable way?** | **+** | **+** | **+** | **+** | **+** | **+** | **+** | **+** | **+** | **+** | **+** | **+** | **+** | **+** |
| **4.Were objective, standard criteria used for measurement of the condition?** | **+** | **+** | **?** | **+** | **+** | **+** | **+** | **+** | **+** | **+** | **+** | **+** | **+** | **+** |
| **5.Were confounding factors identified?** | **-** | **-** | **-** | **-** | **+** | **?** | **+** | **+** | **+** | **+** | **+** | **+** | **+** | **+** |
| **6.Were strategies to deal with confounding factors stated?** | **-** | **-** | **-** | **-** | **?** | **+** | **+** | **+** | **+** | **-** | **-** | **-** | **-** | **-** |
| **7.Were the outcomes measured in a valid and reliable way?** | **+** | **+** | **+** | **?** | **+** | **+** | **+** | **+** | **+** | **+** | **+** | **+** | **+** | **+** |
| **8.Was appropriate statistical analysis used?** | **+** | **+** | **?** | **+** | **+** | **+** | **+** | **+** | **+** | **+** | **+** | **+** | **+** | **+** |
| **% of +** | **62.5** | **50** | **50** | **62.5** | **87.5** | **87.5** | **100** | **100** | **100** | **75** | **87.5** | **62.5** | **75** | **87.5** |
| **Quality of the study** | **MODERATE QUALITY** | **MODERATE QUALITY** | **MODERATE QUALITY** | **MODERATE QUALITY** | **HIGH QUALITY** | **HIGH QUALITY** | **HIGH QUALITY** | **HIGH QUALITY** | **HIGH QUALITY** | **MODERATE QUALITY** | **HIGH QUALITY** | **MODERATE QUALITY** | **HIGH QUALITY** | **HIGH QUALITY** |

**Supplemental Table 2.** Methodological quality of the Cross-Sectional Studies

+=Yes; -=No; ?=“Unclear”; N/A: not applicable
